# Medicine in Ancient Egypt and Ethiopia

**Published:** 1922-03

**Authors:** M. B. Ray


### A FEW NOTES ON MEDICINE IN ANCIENT EGYPT AND ETHIOPIA.

By M. B. RAY, D.S.O., M.D.

THE medicine practised in Ancient Egypt seems to have been a curious combination of superstition and common sense. One of the most prominent reasons for illness the ancient Egyptians thought—and no doubt wisely thought—arose from overeating, and in consequence from indigestion. Three days a month, we read, they underwent some form of cure and also had recourse very often to fasting and to other remedies, such as emetics, purgatives and diuretics. Some herbs found in the desert and still known to the Arabs were used for medicinal purposes. The Egyptians thought themselves the first to invent the cure of diseases; Papyri have been found which contain many medical prescriptions for a variety of diseases, viz., those of the stomach, for broken bones, for the eyes, for sores, and for various other ills, and even to keep away fleas and reptiles. These remedies were often quite good and successful, although they occasionally were rather apt to be akin to the illness to be cured.

An interesting feature of ancient Egyptian medicine was that there was a special doctor for every complaint and he was not allowed to treat any other, so that there was one special physician for the stomach, another for the head, another for the eyes, and so on. It will therefore be seen that medical specialism as understood in these days is by no means new. The Egyptians thought that every part of the body was ruled over by a special god; and this may have been their reason for only allowing each physician to have the cure of one branch. The Egyptians divided the body into thirty-six parts. Their ideas, of course, were exceedingly elementary and crude. From a collection of ancient receipts, we gather that they did not know much more about the human body than that "the breath entering the nose goes to the heart and lungs"; that "the pulse is felt in all parts of the body"; and that "there are vessels going from the heart to all parts." Some gynæcological and veterinary prescriptions were found among them. In the early days the Egyptians appear to have been very little troubled with gouty tendencies.

The actions of the prescriptions were often supposed to be assisted by spells. Origen mentions that when any part of the body was afflicted, they invoked the god (or demon) to whom it belonged to obtain a cure. They also offered *ex votos* in temples which were supposed to help the cure, and after the cure had been made they hung up in the temple a model of the limb or other part which had been healed, so that models of distorted legs or arms, or of an eye, or of an ear, might have been seen there suspended. Dreams were also much credited, and the prayers of the good were thought to be blessed by the remedy for their illness being vouchsafed them.

Both medicine and surgery appeared at an early date in Egypt. The second King of the country, named Athothes, is said to have written a book on anatomy. We are also told that Hermes wrote six medical books and that the first of these was on the subject of anatomy. The four deities chiefly concerned with healing were Thoth, Ptah, I-em-hotep, and Ta-urt. Thoth, one of the first to be connected with healing, is supposed to have given wisdom to doctors. He was thought to have been the inventor of magic and of science. The Greeks identified him with Hermes. At a later period, the name of "the Hermetic art" was given by alchemists to their work, and the phrase we now so often use, "hermetically sealed," no doubt comes down to us from the seal of Hermes, which they placed on their vessels. In early monuments we see Thoth represented by an ibis, and his statue has the head of that bird. Near the city where he was worshipped, Hermopolis Magna, a great number of these birds, which were regarded as being sacred to him, are buried.

Ptah, the chief God of Memphis, was another God of healing. He was mostly concerned with the curing of deafness and blindness. He is called "Father of the Mighty Fathers" and "The Creator of his own image." Besides being a God of healing, he was known as a great artist and builder. The bull of Apis—a black bull with white spots on it—was regarded as a reincarnation of Ptah. Ptah is represented often as a shrouded figure with a sceptre and the Sign of Life, and is depicted as standing on a pedestal within a shrine.

I-em-hotep, who lived during the third dynasty was a most learned physician. Probably also he was a priest of Ra—the Sun-God. He was deified as the special god of medicine; this came about during the course of ages, and because he had founded such an exceedingly eminent cult. He healed folk, and is described as being both merciful and kind, giving sleep to the suffering, relieving pain and curing diseases. After he was raised to be a demi-god he became one of the triad of Memphis. The cult of the medicine-god originated in that city, where a large temple was dedicated to him. One of his titles is "the good physician of gods and men." He is also called the creative god, and is said to have given life to all men and sons to the childless. He and his assistant priests gave advice and medical treatment to vast numbers of sick folk. The priests went on with the work of healing after his death, and did so in his name. Later on, temples of a like nature were erected elsewhere and the priest-physicians moved from Memphis to other places. The Greeks seem to have regarded I-em-hotep as being like the Greek Asklepios, for they called him Asklepieia. This was when the Greek colonists came to Egypt in later days.

I-em-hotep and his priest-physicians treated some of the diseases which are known to us at the present day, such as anæmia, tubercle, plague and leprosy. Besides being a physician he was many other things—an architect, a minister to the King, an astronomer, an alchemist and a writer. He was clever in all these things, but cleverest of all in medicine. Some elementary facts about the movement and circulation of the blood most likely came through his researches, or perhaps through those of other priest-physicians. Anyway the Egyptians knew as much as did the Greeks on these matters, and it may very well be that the Greeks derived their knowledge from the Egyptians. The priest-physicians often performed necropsies, the examination of the dead body being made to find out the cause of death. We do not know whether they discovered much ; no doubt they did obtain a partial knowledge of the circulation.

The goddess Ta-urt was the patroness of pregnant women and one of the goddesses of child-birth. She was in attendance when gods and when kings were born. Ta-urt was the wife or concubine of Set, and she figures in the cult of the dead, for she was Guardian of the Mountains of the West through which the road to the Underworld lay. Some of her titles were : " Bearer of the Gods," " Goddess of Protection," and " Lady of Heaven " and " Mistress of All the Gods." She was known as the hippopotamus goddess and is represented as standing on her hind legs. Her paunch is distended, and her hands or fore-feet nearly always rest upon her characteristic emblem, the *Sa*, the sign typical of protection. The *Sa* sign is the emblem of the magical fluid of life or of the protective magical power the goddess exercised over the female organs. Ta-urt is depicted as having the breasts of a woman and at times as having also the hands of a woman. Occasionally she is represented with a woman's head, or the head of a lioness or that of a bear. Her image, made of earthenware, was worn as an amulet and at child-birth it was worn as a prophylactic. A figure of Ta-urt was sometimes worn by children to protect them.

There is a sculptured tablet at the temple of Kôm-Ombos (which is situated on the Nile and is 550 miles or so from Cairo), composed of representations of instruments concerned with gynæcology. Where the temple now stands there stood formerly the ancient city of Pa-Sebek, where the Crocodile God was worshipped, and the hawk-headed Horus, called Heru-Ur, or the Greater Horus, the Aroueris of the Greeks. This great temple was built by Ptolemy VII., but there were traces of an earlier building, built by Thotmes III. and dedicated to the god Sebek, dating from 1600 B.C. The temple was built on a wide terrace on the river bank. A small temple and a propylon were on this terrace, built about A.D. 83 by the Roman Emperor Domitian, whose cartouche appears on the walls.

Near to the temple of Domitian are the ruins of a small building of a sacred character which appears to have been built for Ptolemy IX. about the year 140 B.C. It seems to have been one of those structures of a religious architecture, peculiar to Egypt and called the " Birth House," or Mammisi, and was a building which commemorated the " Divine Birth of the King." The Mammisis which we find at Kôm-ombos and at Philæ, Abydos, Edfu, and Esneh were Ptolemaic or Roman in character, though they can be traced back in origin to representations in the Egyptian temples of 1600 B.C. Representations of a like character are to be seen on the walls of the Theban Temples of Deir-el-Bahari, built by Queen Hatshepsu (1600 B.C.), and of Amenophis III. at Karnak. These scenes show the accouchement of the Queen, attended by the Hathors, and the appearing of the god Amen-Ra in human form to the Queen-mother. The Ptolemaic Birth House belongs to a period of very confused ideas in Egypt regarding religion, and these ideas were often mixed up with Greek symbolism. They are in consequence difficult to explain. In the Birth House at Kôm-Ombos there is represented a seated deity who forms the central figure. The head is missing, but from the pieces of sculpture remaining near by, it may with good reason be thought to be a representation of the hawk-headed Heru-Ur. Behind this figure is standing a female deity, with one hand held up and in the other hand the Symbol of Life. This, no doubt, is meant for Isis, the inscription which is in the centre of the tablet being addressed to her. On the altar in front of the seated deity there is an upright panel divided into four compartments. This panel is probably meant to represent a surgical cabinet which contains a number of gynæcological instruments in use in Ptolemaic times.

Very little has come down through the ages with regard to the practice of medicine in Ethiopia. At one time Egyptian civilisation extended into Nubia. It, however, gradually declined. We know that shortly after 730 B.C. an Ethiopian dynasty (the twenty-fifth) established itself firmly on the Egyptian throne, but it only lasted a little over a century, as the Ethiopian kings were forced to give way before the Assyrians and their kingdom was restricted to Nubia, the northern border of which lies near Philæ. We know that about 600 B.C. the royal residence of the kings of Ethiopia was established at Meroë, which lies much farther to the south. We also know of an unsuccessful campaign of the army of Psammetikh II. against Lower Nubia about 590 B.C., and of the attempts of Cambyses (525 B.C.) to conquer Ethiopia. After that, an almost impenetrable veil falls over the history of the country. We may assume that the medical ideas obtaining in Egypt would also penetrate into Ethiopia, because the bulk of our information regarding ancient Ethiopia is derived from Egyptian monuments. The employment of the Geez or Ethiopic language for literary purposes did not begin until very nearly the Christian era. As a literary language it is considered to have survived its use as a vernacular. The only known scientific treatise is a medical one, of which the British Museum possesses a copy. During the last decade some highly interesting excavations have been made in various sites in ancient Ethiopia and many pathological and other findings brought to light.

---

Mr. R. J. Coles, the secretary of King's College Hospital, lately told us the story of two small Londoners whom he saw stopping to read the notice on the Hospital railings, " Compelled to close 160 beds owing to lack of funds." " The Horspital's closing !" said one, with evident concern, and both, without further discussion, dropped several coppers into the collecting-box. Many people must have been impressed by this charming tale of juvenile generosity and public spirit. A recent donor of £100 to the funds of the hospital told Mr. Coles that it had inspired his gift, and expressed the hope that it might influence others in the same way.

